# Retrograde intrarenal surgery with or without ureteral access sheath: a systematic review and meta-analysis of randomized controlled trials

**DOI:** 10.1590/S1677-5538.IBJU.2024.0452

**Published:** 2024-08-20

**Authors:** Lucas Guimarães Campos Roriz de Amorim, Marcelo Esteves Chaves Campos, Lígia Sant’Ana Dumont, José Augusto Rojas Peñafiel, Eliabe Silva de Abreu, Giovanni Scala Marchini, Manoj Monga, Eduardo Mazzucchi

**Affiliations:** 1 Universidade Federal de Minas Gerais Departamento de Urologia Belo Horizonte MG Brasil Departamento de Urologia, Universidade Federal de Minas Gerais, Belo Horizonte, MG, Brasil; 2 Universidade Evangélica de Goiás Departamento de Medicina Anápolis GO Brasil Departamento de Medicina, Universidade Evangélica de Goiás, Anápolis, GO, Brasil; 3 SEK International University Department of Medicine Quito Ecuador Department of Medicine, SEK International University, Quito, Ecuador; 4 Mayo Clinic-Rochester Department of Medicine Rochester MN USA Department of Medicine, Mayo Clinic-Rochester, Rochester, MN, USA; 5 Hospital das Clínicas da Faculdade de Medicina da Universidade de São Paulo Departamento de Urologia São Paulo SP Brasil Departamento de Urologia do Hospital das Clínicas da Faculdade de Medicina da Universidade de São Paulo, São Paulo, SP, Brasil; 6 UC San Diego Health Department of Urology La Jolla CA USA Department of Urology, UC San Diego Health, La Jolla, CA, USA

**Keywords:** Urolithiasis, Meta-Analysis as Topic, Surgical Procedures, Operative

## Abstract

**Introduction:**

The ureteral access sheath (UAS) is a medical device that enables repeated entrance into the ureter and collecting system during retrograde intrarenal surgery (RIRS). Its impact on stone-free rates, ureteral injuries, operative time, and postoperative complications remains controversial. Therefore, we performed a systematic review and meta-analysis comparing RIRS with versus without UAS for urolithiasis management.

**Purpose:**

To compare outcomes from retrograde intrarenal surgery (RIRS) for stone extraction with or without ureteral access sheath (UAS); evaluating stone-free rate (SFR), ureteral injuries, operative time, and postoperative complications.

**Materials and Methods:**

We systematically searched PubMed, Embase, and Cochrane Library in June 2024 for randomized controlled trials (RCTs) evaluating the efficacy and safety outcomes of UAS use in RIRS for urolithiasis treatment. Articles published between 2014 and 2024 were included. Pooled risk ratios (RRs) and mean differences (MDs) were calculated for binary and continuous outcomes, respectively.

**Results:**

Five RCTs comprising 466 procedures were included. Of these, 246 (52.7%) utilized UAS. The follow-up ranged from 1 week to 1 month. UAS reduced the incidence of postoperative fever (RR 0.49; 95% confidence interval [CI] 0.29–0.84; p=0.009), and postoperative infection (RR 0.50; 95% CI 0.30–0.83; p=0.008). There were no significant differences between groups in terms of SFR (RR 1.05; 95% CI 0.99–1.11; p=0.10), ureteral injuries (RR 1.29; 95% CI 0.95–1.75; p=0.11), operative time (MD 3.56 minutes; 95% CI −4.15 to 11.27 minutes; p=0.36), or length of stay (MD 0.32 days; 95% CI −0.42 to 1.07 days; p=0.40).

**Conclusion:**

UAS leads to a lower rate of post-operative fever and infection. However, UAS did not significantly reduce or increase the SFR or the rate of ureteral injuries during RIRS for patients with urolithiasis. The use of UAS should be considered to decrease the risk of infectious complications, particularly in those who may be at higher risk for such complications.

## INTRODUCTION

The ureteral access sheath (UAS) is a medical device used to guide and facilitate the passage of the scope and improve visualization during retrograde intrarenal surgery (RIRS) for kidney stone management. The UAS facilitates multiple entries into the ureter and collecting system, reduces intrarenal pressure, and preserves the scope during stone extraction. UAS may additionally preserve the ureteral mucosa since it prevents direct contact between the scope and the mucosal lining (1). However, transient ureteral ischemia and the risk of ureteral injuries potentially increases the risk of postoperative ureteral stricture and obstruction (2), which contributes to the remarkable controversy regarding the routine utilization of UAS during RIRS.

Several primary studies and systematic reviews have addressed the efficacy and safety of RIRS with versus without UAS (1, 3–5). However, they included observational data, which may have introduced bias and confounding factors and led to less generalizable findings. Due to the scarcity of high-level evidence, most recommendations in international guidelines are based on a non-randomized prospective cohort (6). Considering this limitation and the recent release of key randomized controlled trials (RCTs), we aimed to conduct an updated systematic review and meta-analysis restricted to RCTs comparing the outcomes of patients undergoing RIRS with versus without UAS to provide more reliable and updated evidence, thereby enhancing internal validity, reducing the risk of bias and reinforcing the importance of this study in guiding urologic practice.

## MATERIAL AND METHODS

Our study was conducted and reported in accordance with the Cochrane Handbook for Systematic Reviews of Interventions and Preferred Reporting Items for Systematic Reviews and Meta-Analysis (PRISMA) Statement guidelines (7, 8). The study was prospectively registered in the International Prospective Register of Systematic Reviews (PROSPERO) database under protocol CRD42023429216.

### Eligibility criteria

Inclusion in this meta-analysis was restricted to studies that met all the following eligibility criteria: RCTs; published between 2014 and 2024; comparing the RIRS approach with versus without UAS to manage kidney or proximal ureteral calculi; and reporting any of the outcomes of interest. We excluded studies lacking a control group; evaluating mid or lower ureteral calculi; unpublished full-text articles (conference abstracts); and preliminary results from published RCTs.

### Search strategy

We systematically searched PubMed, Embase, and Cochrane Central Register of Controlled Trials in June 2024 for studies that met our inclusion criteria. Articles published between 2014 and 2024 were included. The following medical subject heading terms were included for a Medline search and adapted for other databases as needed: ("ureteral access" OR "ureteric access" OR ureteroscopy OR ureteroscopic OR "retrograde intrarenal" OR "retrograde intra renal" OR "retrograde intra-renal" OR ureterorenoscopy) AND (RCT OR random OR randomization OR randomly OR randomized). There were neither language nor patient population size restrictions for the search.

All identified articles were systematically assessed using the above-cited prespecified criteria. Two authors independently performed screening and selection of studies (L.A. and L.D.). Disagreements were resolved through consensus among the authors.

### Data extraction and missing data

Two authors (L.A. and L.D.) independently extracted data from the selected studies utilizing a standardized data extraction sheet. The authors resolved disagreements through consensus. We requested relevant missing or potentially inconsistent information from the selected studies by email to the authors.

### Endpoints and definitions

The intraoperative endpoints of interest were operative time, and ureteral injuries. The postoperative endpoints of interest were stone-free rates (SFR), length of stay (LOS), postoperative fever, and postoperative infection.

Among the studies, the SFR outcome was defined as having residual fragments measuring < 3 or 4 millimeters. The follow-up time at which residual fragments were evaluated (at 3, 7, 14, or 30 days), as well as the imaging method used for assessment (radiography, ultrasonography, or computed tomography scan), varied among studies, as detailed in Table-1.

**Table 1:** Individual characteristics of studies and their SFR assessments.

Study	Procedures (n)	Age (years)*	Male (%)	BMI (kg/m^2^)*	Stone burden (mm or mm^2^)*	Stone location (%)	Follow-up	SFR definition	SFR imaging method	SFR time assessment (POD)
Abdelfatah Zaza et al., 2023 (11)	33/31	43.8/42.7	64/58	29/28.7	16.8/16.5 mm	Upper pole: 33.3/32.3 Mid pole: 30.3/25.8 Lower pole: 21.2/25.8 Renal pelvis: 12.1/9.7 Upper ureter: 3.0/6.5	1 week	CIRF <4 mm	NC-CT KUB	7
Bozzini et al. 2024 (12)	92/89	51.4/48.3	44/47	NA	15.8/14.1 mm	NA	2-4 weeks	CIRF <3 mm	CT KUB	3
Ecer et al. 2022 (13)	40/20	47.1/50.5	72/65	28.6/29.8	13.6/14.9 mm	Upper pole: 7.5/10 Mid pole: 22.5/15 Lower pole: 22.5/25 Renal pelvis: 17.5/25 Multiple: 30/25	2 weeks	CIRF <3 mm	US and/or NC-radiography (previously diagnosed with abdominopelvic CT)	14
Singh et al. 2023 (14)	41/40	38.9/39.1	78/57	26.7/26.7	14.7/15.33 mm	Lower pole: 39/37.5	1 month	CIRF <3 mm	NC-CT KUB	30
Turan et al. 2024 (15)	40/40	48.8/48.5	75/72.5	25/25.3	139/141 mm2	Upper pole: 10/12.5 Mid pole: 25/30 Lower pole: 30/25 Renal pelvis: 35/32.5	1 month	CIRF <4 mm	NC-CT KUB	30

Values refer to groups with/without ureteral access sheath;

* mean or median; BMI = body mass index;

CIRF = Clinically insignificant residual fragments; CT = computed tomography; KUB = kidneys, ureters and bladder; NA = not available; NC = non-contrast; POD = postoperative days; SFR = stone-free rate; US = ultrasound.

The diagnosis of postoperative symptomatic urinary tract infection was defined based on patient-reported symptoms, physical examination, postoperative fever (>38°C), postoperative urosepsis, bedside dipstick urinalysis, or urine culture.

In each study, urologists closely observed the final endoscopic passage exiting the ureter to evaluate postoperative ureteral injuries. The lesions were graded based on the Post-Ureteroscopic Lesion Scale (PULS) grading system (9). This scale categorizes lesions into six groups. Grade 0 indicates no lesions or insignificant abrasions, while grade 1 represents superficial mucosal lesions, significant mucosal edema, or hematoma. Grade 2 signifies submucosal lesions without contrast media extravasation. Grade 3 denotes perforation with less than 50% (partial) transection and contrast media extravasation. Grade 4 corresponds to perforation with more than 50% but less than 100% (partial) transection. Grade 5 indicates complete transection. Lesion grading remains independent of their location or extent. The severity of the most significant lesion determines the overall PULS grading in cases involving multiple lesions.

### Quality assessment

We used the Cochrane Collaboration's tool for assessing the risk of bias in randomized trials (RoB 2) for quality assessment of RCTs (10). Three authors independently conducted the risk of bias evaluation (L.A., L.D., and J.P.). The authors resolved disagreements through consensus. Small study effects (publication bias) were assessed through funnel plot analysis for the outcome of SFR (main outcome) and evaluation for a symmetrical distribution of trials with similar weights. We also performed a leave-one-out sensitivity analysis to assess whether the results largely relied on a single study.

### Statistical analyses

Treatment effects for binary endpoints were computed using pooled risk ratios (RRs) with 95% confidence intervals (CIs), whereas continuous endpoints were computed using mean differences (MDs) with 95% CIs. The Mantel‒Haenszel statistical effect model was utilized for all binary endpoints, while the inverse-variance method was applied for continuous endpoints using the DerSimonian Laird random-effects model. Heterogeneity was assessed through I^2^ statistics, and prediction intervals. Review Manager version 5.4.1 (Nordic Cochrane Centre, The Cochrane Collaboration, Copenhagen, Denmark) was used for statistical analyses, and the Comprehensive Meta-Analysis Prediction Intervals Program was utilized for the calculation of the prediction intervals.

## RESULTS

### Study selection and baseline characteristics

**Figure 1:**
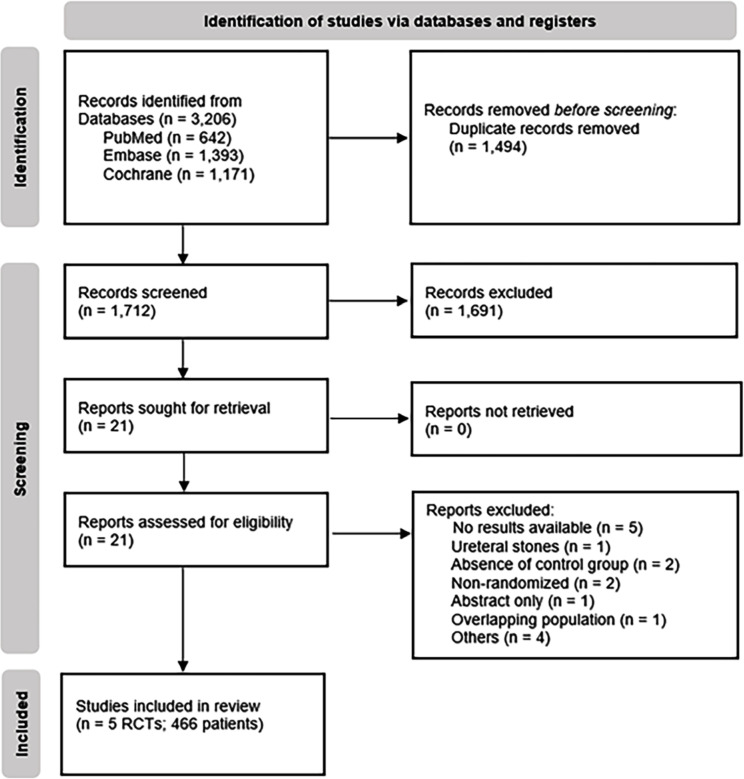
PRISMA flow diagram of study screening and selection.

As detailed in Figure-1, our initial search yielded 3,206 results. After the removal of duplicate records and ineligible studies, 21 remained and were fully reviewed based on prespecified criteria. Of these, a total of five RCTs, published between 2021 and 2024, were included. These trials encompassed 466 procedures, of which 246 (52.7%) were performed with UAS (11–15).

RCTs, randomized controlled trials.

Individual study characteristics are reported in Table-1. Most patients were male (59.6%). The mean age ranged from 38.9 to 51.4 years, with a mean follow-up duration ranging from 1 week to 1 month. Four studies limited their inclusion criteria to renal stones, while one study also included 3 patients (4.6%) with upper ureteral stones (11).

RIRS was performed using flexible scopes across all patients. In two studies, an 8-Fr rigid or semi-rigid ureteroscopy preceded UAS placement (13, 15). Notably, one of these studies presented two patient cohorts using UAS: the standard UAS (STUAS) group and dual-lumen UAS (DLUAS) group (13). The DLUAS is a vacuum-assisted sheath, a single lumen sheath with an oblique side designed for connection to a vacuum system for active drainage. However, it is noteworthy that in this study, the DLUAS was not connected to a suction apparatus and functioned similarly to a standard UAS (16). Therefore, we aggregated data from both cohorts into our ‘with UAS’ group for statistical analysis using Review Manager 5.4.1.

Additionally, in one study, all patients underwent preoperative stenting with double-J at least 10 days before the procedure and received postoperative Tamsulosin 0.4 mg once daily until removal of the postoperative stent, which occurred 2 weeks after the procedure (and 2 weeks before assessing SFR) (14). The procedural characteristics of the included studies are detailed in Table-2.

**Table 2:** Procedural characteristics of the included studies.

Study	Abdelfatah Zaza et al. 2023 (11)	Bozzini et al. 2021 (12)	Ecer et al. 2022 (13)	Singh et al. 2023 (14)	Turan et al. 2024 (15)
UAS Size, (Fr)	NA	10-12	11-13	9.5-11.5	9.5-11.5
Prior URS before UAS placement	NA	No previous URS	Cystoscopy followed by 8-Fr rigid URS	NA	8-Fr semi-rigid URS
Preoperative ureteral dilatation type	NA	No preoperative dilatation	No preoperative dilatation	Preoperative stenting with DJ at least 10 days prior procedure	NA
Postoperative stent, n (%)	NA	92(100)/89(100)	39(97.5)/19(95)	41(100)/40(100)	40(100)/40(100)
Postoperative stent type	NA	6 Ch DJ, removed in 14-28 days	DJ, removed in 14 days if possible	4.8-Fr DJ, removed in 14 days; or per urethral catheter	DJ
Fragmentation device	NA	272μm Ho:YAG	200μm Ho:YAG 10 Hz/2.5 J	365μm Ho:YAG 10 Hz/1 J	Ho:YAG
Basketing or Grasping	NA	Kobot Filter basket to retrieve some fragments	NA	Basket or tri-prong flexible forceps to relocate inferior calyx stones	NA
Irrigation	NA	Gravity irrigation supplemented with on-demand Traxer Flow® dual port flushing	Gravity irrigation (kept at 60cm height)	Path finder saline irrigation (kept at 40cm height)	Gravity irrigation (kept at 100 cm height)

Values refer to groups with/without UAS; DJ = double-J ureteral stent; Ho:YAG = Holmium Yttrium Aluminium Garnet fibre laser; NA = not available; UAS = ureteral access sheath; URS = ureteroscopy

### Pooled analysis of all studies

**Figure 2:**
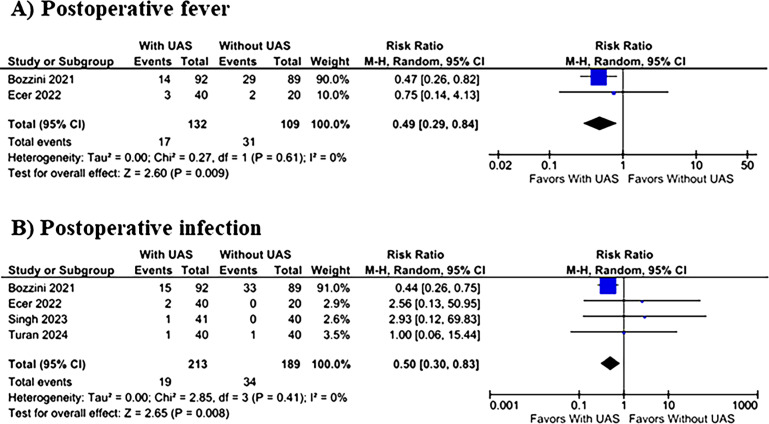
Incidence of (A) postoperative fever and (B) postoperative infection in UAS x Without UAS groups.

**Figure 3:**
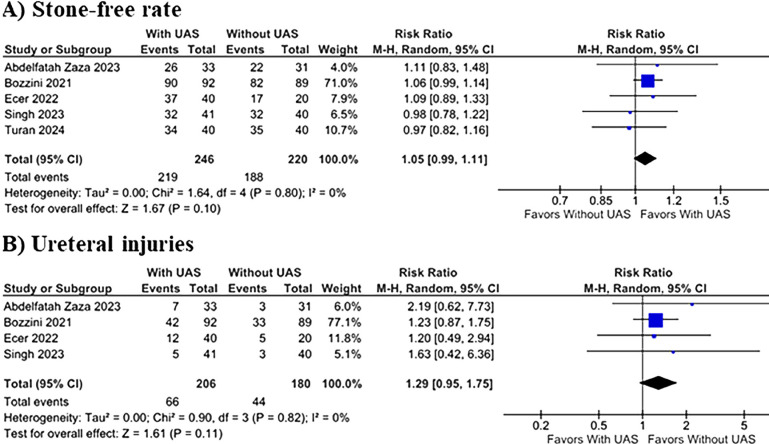
The (A) SFR and the rate of (B) ureteral injuries in UAS x Without UAS groups.

The use of UAS during RIRS significantly reduced the incidence of postoperative fever (12.8% vs. 28.4%; RR 0.49; 95% CI 0.29–0.84; p=0.009; I^2^=0%; Figure-2A), and postoperative infection (8.9% vs. 17.9%; RR 0.50; 95% CI 0.30–0.83; p=0.008; I^2^=0%; Figure 2B). There was no difference between groups in terms of SFR (89.0% vs. 85.4%; RR 1.05; 95% CI 0.99–1.11; p=0.10; I^2^=0%; Figure-3A), and ureteral injuries (32.0% vs. 24.4%; RR 1.29; 95% CI 0.95–1.75; p=0.11; I^2^=0%; Figure-3B).

The incidence of (A) postoperative fever (p = 0.009) and (B) postoperative infection (p = 0.008) were significantly reduced in the group with UAS compared to the group without UAS.

CI, confidence interval; df, degrees of freedom; M-H, Mantel-Haenszel; UAS, ureteral access sheath

The (A) SFR and the rate of (B) ureteral injuries were not significantly different between groups.

CI, confidence interval; df, degrees of freedom; M-H, Mantel-Haenszel; SFR, stone free rate; UAS, ureteral access sheath.

**Figure 4:**
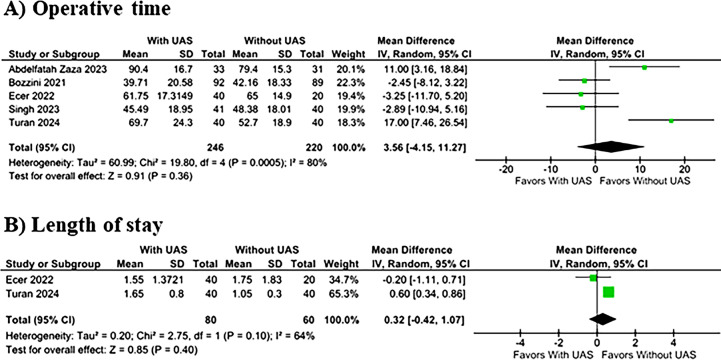
The (A) operative time and (B) LOS in UAS x Without UAS groups.

There were no significant differences between groups in operative time (MD 3.56 minutes; 95% CI −4.15 to 11.27 minutes; p = 0.36; I^2^ = 80%; Figure-4A) or LOS (MD 0.32 days; 95% CI −0.42 to 1.07 days; p = 0.40; I^2^ = 64%; Figure-4B).

The (A) operative time and (B) LOS were not significantly different between groups.

CI, confidence interval; df, degrees of freedom; IV, inverse-variance; SD, standard deviation; UAS, ureteral access sheath.

### Quality assessment

**Figure 5:**
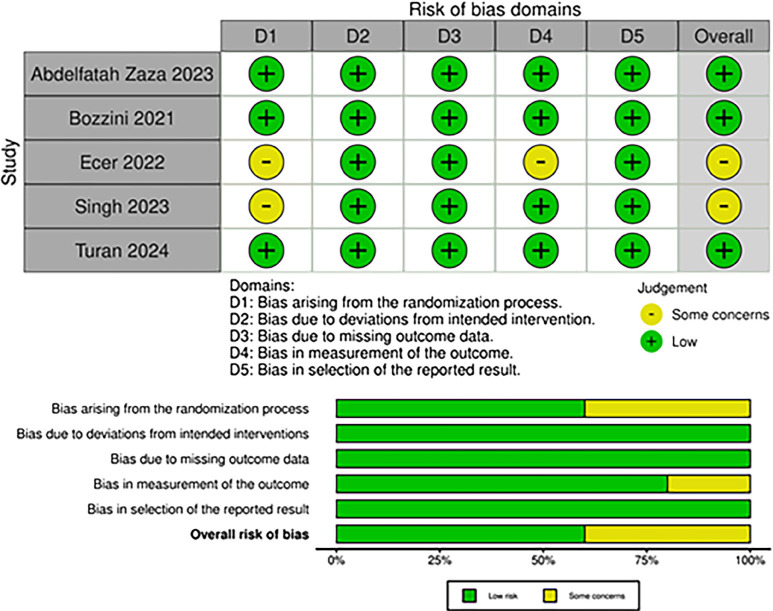
Risk of bias assessment using RoB 2 tool.

Individual RCT appraisals, performed as per the Cochrane Collaboration's RoB2 tool, are reported in Figure-5. One study was deemed to be of some concern for not using computed tomography (CT) scans to evaluate SFR, while another study was rated as having concerns due to a significant difference in male prevalence between groups (13, 14).

Ecer's study was deemed to be of some concern for not using computed tomography (CT) scans to evaluate SFR, while Singh's was rated as having concerns due to a significant difference in male prevalence between groups. Overall, no major concerns were observed regarding the quality of the studies individually.

**Figure 6:**
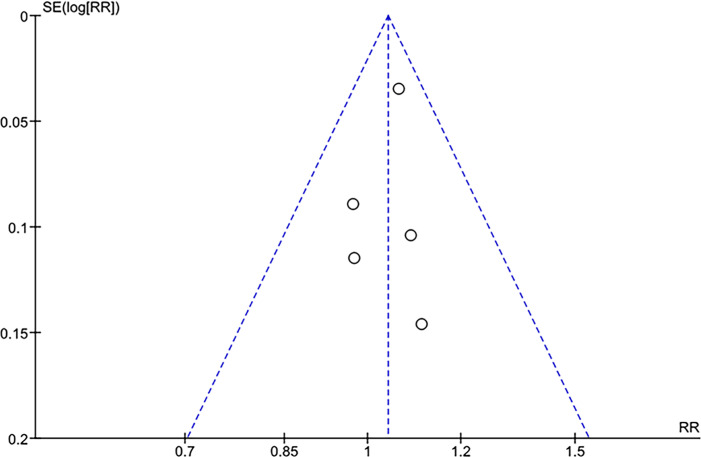
Funnel plot for the SFR outcome.

A funnel plot analysis for the outcome of SFR revealed no evidence of small study effects (publication bias), as reported in Figure-6. Studies exhibited a symmetrical distribution according to weight and converged toward the pooled effect as weight increased. Egger's regression test could not be performed due to the limited number of included studies (n < 10).

A funnel plot analysis for the outcome of SFR revealed no evidence of small study effects (publication bias). Studies exhibited a symmetrical distribution according to weight and converged toward the pooled effect as weight increased. RR = risk ratio; SE = standard error; SFR = stone free rate

### Sensitivity analyses

Overall, sensitivity analyses using the leave-one-out approach revealed consistent results compared with the pooled analysis of all studies when individual studies were sequentially excluded from the analysis for the outcomes of SFR, ureteral injuries, and operative time. This approach could not be employed in postoperative fever and LOS due to the limited number of studies included in these outcomes analyses.

Although exhibiting null heterogeneity in the pooled analysis, the postoperative infection leave-one-out sensitivity analysis revealed that its results were driven mostly by one study, probably due to its elevated weight (12). When excluding this study, no significant difference was found between groups.

**Figure 7:**
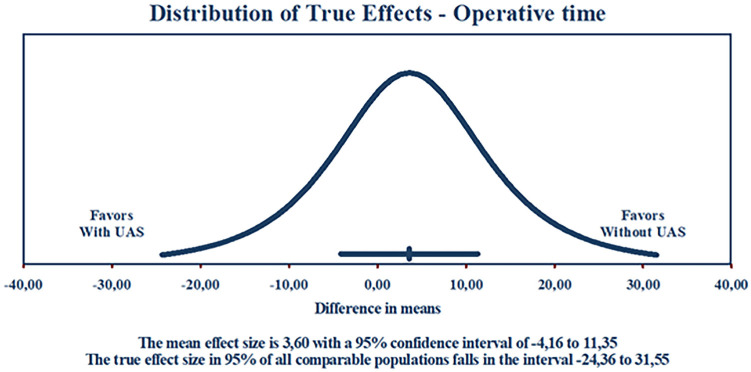
Prediction interval for the operative time outcome.

The binary endpoints exhibited null heterogeneity. In contrast, the outcomes of operative time, and LOS had elevated heterogeneity, with I^2^ values of 80% and 64%, respectively. In the assessment of operative time, considering that the true MDs within the universe of comparable populations follow a normal distribution, we can estimate a 95% prediction interval for MDs to range from −24.36 to 31.55 minutes, as illustrated in Figure-7. Due to the limited number of included studies (n < 10), further meta-regression analyses were not feasible.

UAS = ureteral access sheath

## DISCUSSION

In this meta-analysis of five RCTs and 466 patients comparing RIRS with versus without UAS for the treatment of urolithiasis, our main findings were as follows: the use of UAS was associated with a significant reduction in the postoperative incidence of fever and infection; there was no significant difference between groups in the incidence of ureteral injuries; and the SFR was comparable between RIRS with and without UAS.

A previous meta-analysis evaluating the role of UAS in urolithiasis found no significant differences in SFR, operative time, hospitalization time, or intraoperative complications, while it significantly increased the risks of postoperative complications (5). However, this study relied heavily on observational data, making it susceptible to the influence of confounding factors. To address this limitation and provide more reliable and updated evidence, we restricted inclusion to recently released RCTs (11–15).

By doing so, our study confirmed prior results of comparable outcomes between groups, especially SFR, operative time, LOS, and intraoperative complications, but increased confidence and generalizability given the above-cited methodological improvements. More importantly, our meta-analysis found a significantly lower incidence of postoperative fever and infection in the UAS arm, which has not been demonstrated previously.

UAS is associated with enhanced fluid drainage during RIRS, leading to reduced intrapelvic pressure compared with RIRS without UAS (17, 18). This mechanism potentially explains the observed decrease in postoperative fever and infection rates noted in the group with UAS. Our findings align with a large retrospective study conducted by Traxer et al., which included 2239 patients (67% in the UAS group) and reported significantly lower rates of postoperative fever and infection in the UAS arm, 28,6% and 18,6%, respectively (19).

As the device aids in the visualization of the superior urinary tract and drainage of stone fragments, a greater SFR would theoretically be expected when UAS is used in RIRS. Interestingly, our meta-analysis revealed no significant difference between the groups with and without UAS in this outcome. In fact, a few factors may have a greater impact on SFR than using UAS during RIRS, especially the surgeon's experience, preoperative medical expulsive therapy with an α-blocker one week prior to the procedure, and ureteral stenting placement before the ureteroscopy (20–23).

Besides, new technologies are significantly enhancing kidney stone management across all stages. The use of artificial intelligence can improve detection, reducing the diagnostic time and accelerating decision-making (24). New laser instruments, including high-powered Holmium, which was used as the fragmentation device in all the RCTs included, enhance precision and efficacy in stone elimination (25). However, in cases such as lower pole stones with acute infundibulopelvic angles, hard stones (CT value > 1000), or stones encased in abscess-like material, basketing might still be preferred (26). Additionally, combined approaches have been proposed for stones larger than 2 cm, such as ultrasound-guided endoscopic combined intrarenal surgery (23), which may offer a solution beyond the "either/or" dilemma between percutaneous nephrolithotomy and endoscopic procedures (27).

Ureteral injuries remain the main shortcoming of the literature when using UAS in RIRS. Although the device enables multiple straightforward passages of ureteroscopic instruments through a single insertion, it may cause ureteral mucosal injuries directly (4, 28). Nonetheless, RIRS itself is associated with ureteral trauma, irrespective of UAS (9). Of note, some independent factors may increase the risks of ureteral damage, such as male gender, higher stone burden, difficulty in placing sheaths, longer insertion time, repeated attempts to position the scope, and use of rigid instruments (29, 30). In this sense, our meta-analysis showed no significant differences between groups in the incidences of ureteral injuries, indicating that UAS may improve visualization during ureteroscopic procedures without increasing or decreasing this intrinsic procedural risk.

While the studies included in our analysis did not specifically evaluate the occurrence of ureteral strictures following UAS placement due to the short follow-up period, a prospective study conducted by Stern et al. revealed that the incidence of strictures associated with high-grade ureteral injuries secondary to UAS placement is comparable to that observed in cases without UAS, not resulting in clinically significant outcomes in the long-term (31).

Our study has limitations. First, there were differences among the included RCTs in terms of follow-up time and imaging method used to measure the SFR. Second, our meta-analysis included a limited number of patients and RCTs, due to the scarcity of available randomized research. This potentially diminishes the statistical power to detect significant differences, while impacting the reliability of estimates for between-study variance in the random-effects, summary effects, confidence intervals, and heterogeneity assessments (32). Finally, we observed elevated between-study heterogeneity in operative time and LOS. Nevertheless, the results were consistent after the leave-one-out sensitivity analysis.

## CONCLUSION

In this meta-analysis of 466 patients from RCTs, we compared RIRS for urolithiasis with versus without UAS. Our findings revealed no significant differences between groups in terms of ureteral injury and SFR, albeit the incidence of postoperative fever and infection were substantially reduced in the group with UAS. Hence, UAS should be considered especially in those patients where infectious complications are a significant concern.

## ABBREVIATIONS

CI: = Confidence interval
LOS: = Length of stay
MD: = Mean difference
PULS: = Post-ureteroscopic lesion scale
PRISMA: = Preferred Reporting Items for Systematic Reviews and Meta-Analysis
RCT: = Randomized controlled trial(s)
RIRS: = Retrograde intrarenal surgery
RoB 2: = Risk of bias 2
RR: = Risk ratio
SFR: = Stone-free rate(s)
UAS: = Ureteral access sheath
